# Dynamic spectral cues do not affect human sound localization during small head movements

**DOI:** 10.3389/fnins.2023.1027827

**Published:** 2023-02-03

**Authors:** Glen McLachlan, Piotr Majdak, Jonas Reijniers, Michael Mihocic, Herbert Peremans

**Affiliations:** ^1^Department of Engineering Management, University of Antwerp, Antwerp, Belgium; ^2^Acoustics Research Institute, Austrian Academy of Sciences, Vienna, Austria

**Keywords:** active localization, head rotation, dynamic cues, spectral cues, binaural audio, front-back confusion

## Abstract

Natural listening involves a constant deployment of small head movement. Spatial listening is facilitated by head movements, especially when resolving front-back confusions, an otherwise common issue during sound localization under head-still conditions. The present study investigated which acoustic cues are utilized by human listeners to localize sounds using small head movements (below ±10° around the center). Seven normal-hearing subjects participated in a sound localization experiment in a virtual reality environment. Four acoustic cue stimulus conditions were presented (full spectrum, flattened spectrum, frozen spectrum, free-field) under three movement conditions (no movement, head rotations over the yaw axis and over the pitch axis). Localization performance was assessed using three metrics: lateral and polar precision error and front-back confusion rate. Analysis through mixed-effects models showed that even small yaw rotations provide a remarkable decrease in front-back confusion rate, whereas pitch rotations did not show much of an effect. Furthermore, MSS cues improved localization performance even in the presence of dITD cues. However, performance was similar between stimuli with and without dMSS cues. This indicates that human listeners utilize the MSS cues before the head moves, but do not rely on dMSS cues to localize sounds when utilizing small head movements.

## 1. Introduction

Human sound localization experiments and models have historically predominantly focused on passive localization, i.e., where the head is held still. However, head movements have repeatedly been shown to improve sound localization (Wallach, 1940; Wightman and Kistler, 1999), especially for sources with little or distorted spectral content (Perrett and Noble, 1997b; Kato et al., 2003; Morikawa et al., 2013). This has led to increasing interest in the influence of head movements on the localization performance.

Unfortunately, modeling active sound localization is significantly more complex than its passive counterpart. The three major acoustic cues that humans use during sound localization are the interaural time difference (ITD), interaural level difference (ILD), and monaural spectral shape (MSS) cues (Blauert, 1997). In spatially static listening conditions, i.e., static sound source and without head movements, ITDs and ILDs contribute to the sound localization in the lateral dimension and listener-specific MSS cues are required to achieve sound localization performance in sagittal planes (Majdak et al., 2020). In a dynamic environment, i.e., with moving sources and listeners, the acoustic cues that the auditory system processes change over time, and thus all have a dynamic counterpart: dynamic ITD (dITD), dynamic ILD (dILD), and dynamic MSS (dMSS) cues. In theory this gives the auditory system many acoustic cues from which the source's location can be inferred, but whether this happens in practice is currently a point of debate. Moreover, active listening requires a separate movement model to complement the acoustic model (McLachlan et al., 2021). This demands insights on the vestibular and motor systems and the way that humans coordinate these systems in conjunction with the auditory system.

Due to these added complexities, it is of great interest to simplify auditory modeling wherever possible. For example, we may consider which cues are essential to localization performance and which could be omitted. Modeling dITD is relatively easy due to its near-linear link with the lateral position of the sound (Cox and Fischer, 2015), whereas the link between the dMSS and source direction seems to be more complex and would result in a considerably more complicated localization model. The movement model can also be simplified, e.g., by restricting it to only small head rotations along a single axis. Such small rotations can be approximated as constant velocity, modeling the movements as a simple first-order derivative for the dynamic acoustic cues without having to consider translation or acceleration. Further, small head rotations can be seen as the first step in understanding and modeling complex movement behavior, because any natural head rotation can be decomposed into a sequence of smaller rotations. Outside the context of modeling, small head movements are important to investigate explicitly because of their constant presence in natural listening (Carlile and Leung, 2016), and from an evolutionary standpoint it may be expected that the brain exploits these movements if they provide additional information.

For horizontal movement of sound sources, the minimum audible movement angle (MAMA) is about 2° at velocities up to 15°/*s* and about 8° at 115°/*s*. When motion is restricted to the vertical, MAMAs are substantially larger at all velocities, ranging from 6° to 12° (Saberi and Perrott, 1990). This data suggests that listeners are more sensitive to (dynamic) binaural cues (ITD and ILD) than to (dynamic) spectral cues (MSS), though all three can be perceivable within a head rotation of 10°, depending on velocity. These results quantify movement discrimination, but it is still unclear how this affects localization performance.

The purpose of this study was to investigate the effects of small head movements (up to ±10°) on sound localization for normal hearing subjects, and determine which dynamic cues are responsible for these effects. Based on current evidence, the hypothesis was that dITD is the dominant dynamic acoustic cue. Further, this cue was expected to have an even more profound positive effect in conditions in which spectral cues are not available. Finally, it was also expected that dMSS cues do not affect localization performance. This hypothesis is supported by the findings that dMSS cues during monaural listening (Hirahara et al., 2021) and pitch rotations (i.e., rotations along the interaural axis) (Thurlow and Runge, 1967; Kato et al., 2003) have no effect on sound localization.

## 2. Methods

### 2.1. Subjects

Seven normal-hearing subjects (six female, one male) participated in the experiment. Their absolute hearing thresholds were within the average (±1 standard deviation) of the age-relevant norms (Corso, 1959; Stelmachowicz et al., 1989) within the frequency range from 0.125 to 16 kHz. The age range of the subjects was between 22 and 30 years. None of the subjects were familiar with this type of experiment.

### 2.2. Apparatus

The experiment was conducted in the loudspeaker array studio (LAS) of the Acoustics Research Institute of the Austrian Academy of Sciences in Vienna. The LAS is a semi-anechoic room consisting of 91 speakers (E301, KEF Inc.) distributed over the sphere within the elevation angles from −47° to 90°. The LAS was equipped with a head-mounted display (HMD, Oculus Rift, CV1, Meta Inc.) for the visual presentation of virtual reality (VR) and three infrared cameras for the tracking of the listener within the six degrees of freedom. The HMD was worn in all conditions. The LAS also provides equipment for the measurement of listener-specific HRTFs by inserting microphones (KE 4-211-2, Sennheiser Inc.) into the ear canals and measuring the responses from the individual loudspeakers. The HRTF measurement procedure corresponded to that from Majdak et al. (2010), without the HMD in place.

The LAS was controlled by a computer running a 64-bit Windows 10, equipped with a 8-core, 3.6-GHz CPU (i7-11700KF, Intel Inc.), 16 GB of RAM, and a graphic card with dedicated 8 GB of RAM (GeForce RTX 3070, NVIDIA Inc.). The computer was controlled by custom software framework ExpSuite version 1.1 which provides modules for various types of stimulation (multi-channel *via* loudspeakers, binaural *via* headphones), for controlling visual interfaces *via* VR, for tracking systems, and many other functionalities (Majdak and Mihocic, 2022). The HRTF measurements were run by the ExpSuite application AMT@ARI version 7.0.31. The experiment was run by the ExpSuite application LocaDyn version 0.8. All ExpSuite applications are freely available (Majdak and Mihocic, 2022).

Free-field signals were presented *via* the 91 loudspeakers, each driven by an amplifier (Sonible d:24, sonible GmbH) connected to a computer via sound interface (RME MADIface USB, RME Audio AG). In order to create virtual sound sources appearing from an arbitrary direction, vector-base amplitude panning (VBAP, Pulkki, 1997) was applied. For a requested direction of the virtual sound, three loudspeaker positions surrounding that direction were found and the amplitude gains for those loudspeakers were calculated. VBAP was implemented within the ExpSuite module YAMI100 version 1.3 running within the puredata version 0.49 (Puckette et al., 1996) environment. Note that only static virtual sound sources were used in this experiment.

The binaural signals were presented *via* open headphones (HD-650, Sennheiser Inc.). The spatialization of the binaural virtual sources was done in real-time in the ExpSuite module SOFAlizer for Unity version 1.6 (Jenny et al., 2018) running within the Unity version 2020.3.34f1 environment. The binaural signals were updated in real time by capturing the subject's position and orientation with the tracking system of the head-mounted display. The tracking system provides an accuracy of 0.76 cm (in a sitting position, inside a room, Borrego et al., 2018), and a latency below 6 ms (Becher et al., 2018). The subject's position and orientation were recorded for later analyses.

### 2.3. Stimuli

The acoustic stimulus used in this experiment was a wideband (20–2,0000 Hz) white noise burst, gated with a 5-ms cosine ramp. The duration was 2,000 ms for static listening and was gated off after 10° of head rotation for dynamic listening.

**Figure 1:**
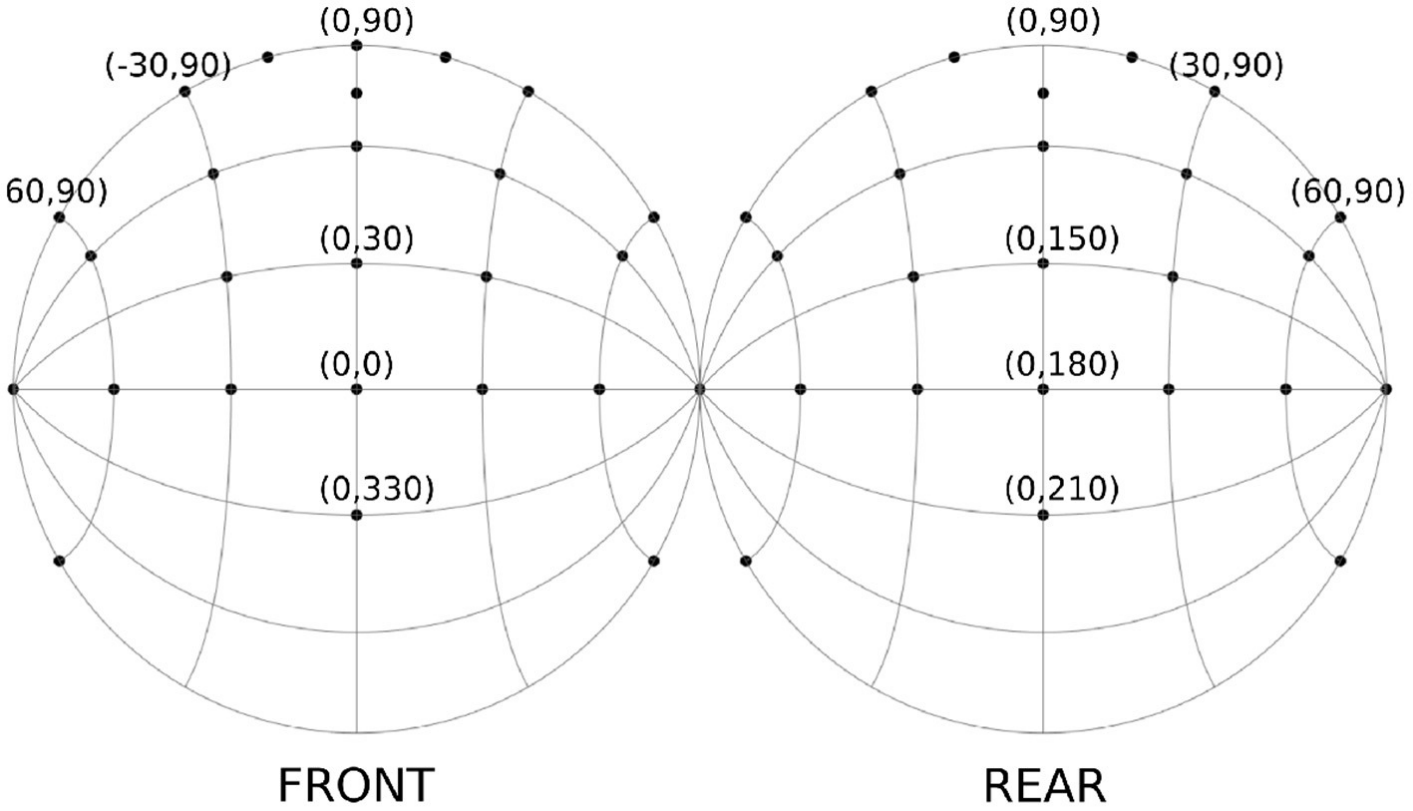
Spatial directions of the sound sources used in the experiment, plotted as two hemispheres cut through the frontal plane. In total 41 directions were used.

Forty-one directions were selected to test the effects of localization over different planes (see Figure 1). These directions were based on the results of a model for dynamic localization (McLachlan et al., 2021) and the results from Perrett and Noble (1997b), which show the largest improvement of errors through head movement for sources around the median plane, especially for sources at high elevations. A higher source density was selected around this area of interest. The sources were further distributed over several sagittal planes, following the so-called “cones of confusion." Because of the left-right symmetry, positions on the midsagittal plane were tested twice, so that the set of tested positions consisted of 52 directions.

**Figure 2:**
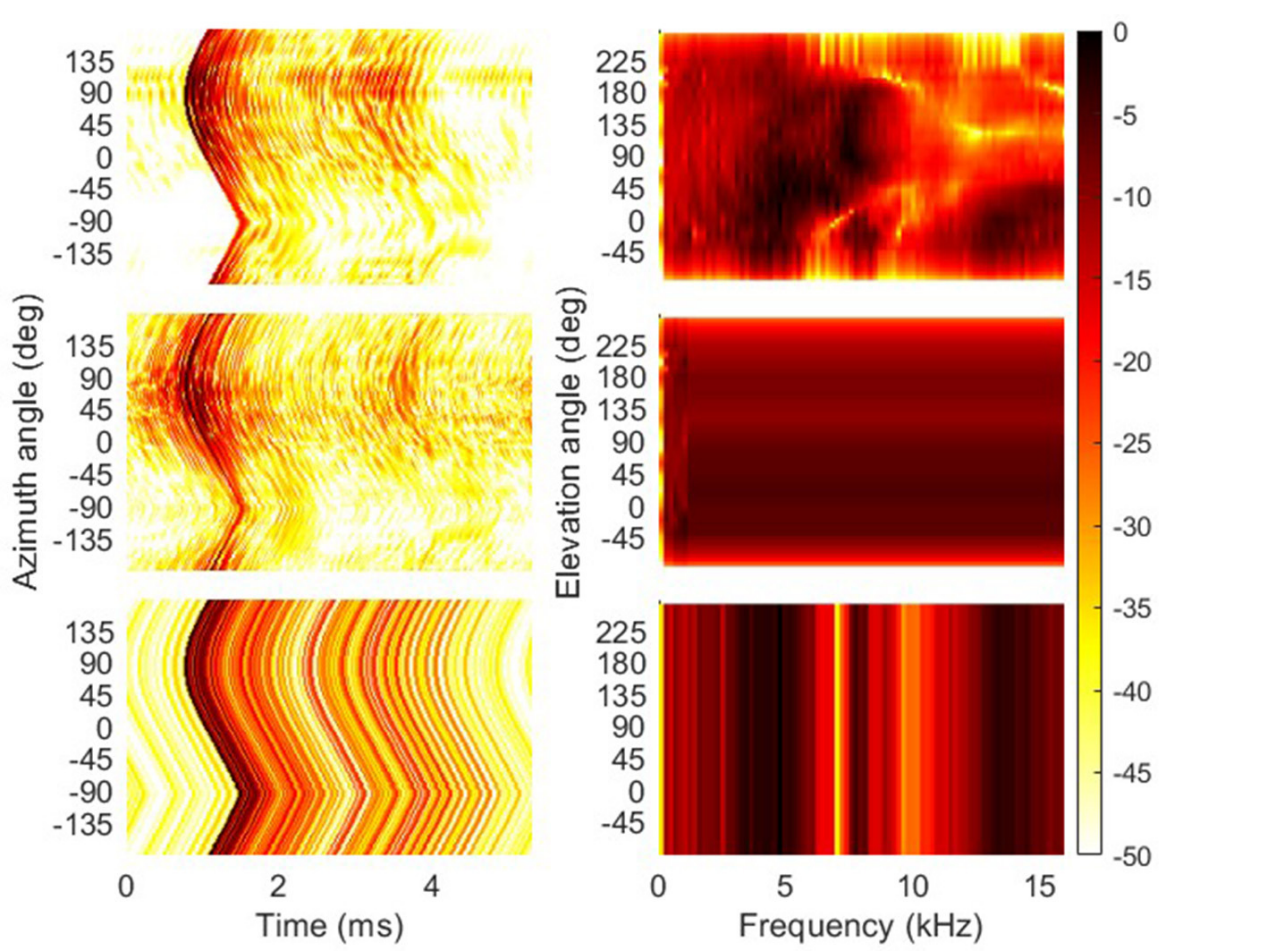
HRTFs of the subject NH257 as an example. **(Left column)** Energy time curves of the impulse responses (in dB) calculated for the HRTFs of a left ear along the azimuth angle. **(Right column)** Magnitude spectra of HRTFs (in dB) along the median plane as a function of the polar angle. **(Top row)** HRTFs from the condition “full." **(Center row)** HRTFs from the condition “flat,” here the MSS was significantly flattened, but the time of arrival, thus, the ITD and dITD cues remained unchanged. **(Bottom row)** HRTFs from the condition “frozen" to (0°,0°), i.e., for all spatial positions, the MSS was frozen to that of the spatial position in the front and at eye level, but the time of arrival, thus, the ITD and dITD cues, were identical to those from the actual spatial positions.

Playback over headphones used listener-specific HRTFs. The acoustically measured HRTFs were available for 91 spatial directions only. Thus, in order to create a smooth real-time dynamic listening environment, each listener-specific HRTF set was interpolated to a denser grid of 5,762 positions. This dense grid was created by subdividing the faces formed by the sparse grid. The subdivision was iterated three times. An HRTF of the dense grid was calculated by removing the time-of-arrival (TOA) by calculating its minimum-phase version, interpolating the spectrum by applying VBAP based on the sparse directions, and introducing a dense TOA modeled by the spherical-head TOA model (Ziegelwanger and Majdak, 2014). Figure 2 shows the acoustically measured and the interpolated HRTFs in the top and center rows, respectively.

### 2.4. Tested conditions

Four acoustic cue conditions were tested, named by their aimed spectral properties: full, flat, frozen and free-field. The first three conditions were run using a virtual acoustic display (VAD), i.e., presented *via* headphones. The full condition used dense listener-specific HRTFs without any further processing. The flat condition used the same HRTFs as in the full conditions, but with flattened frequency-dependent contrasts between 1 and 16 kHz, leaving only (dynamic) ITD and ILD as acoustic cues. This processing was exactly as that in Baumgartner et al. (2017) for *C* = 0. The frozen condition used HRTFs with all cues available while the head was stationary, but considered only ITD changes when the head rotated. Thus, ILD and MSS cues remained static, i.e., frozen to the ILD and MSS for the head in the initial orientation. Figure 2 shows the frozen HRTFs in the bottom row. The free-field condition refers to the loudspeaker-based stimulus presentation using VBAP to create a virtual sound source at the requested direction. This condition served as a sanity check for the VAD results, where, assuming an adequate VAD playback using listener-specific HRTFs, similar results were expected between the free-field and full conditions.

Three types of head rotations were tested: static (i.e., no head movement), yaw rotation, and pitch rotation. The two rotations were single-sided (i.e., from reference position (0°, 0°) toward a specific point). The signal was gated off so that only 10° of the rotation contained acoustic information. The yaw rotation evokes large interaural changes whereas pitch rotation evokes minimal interaural changes but large monaural changes, enabling a clear comparison between the individual dynamic cues.

### 2.5. Procedure

#### 2.5.1. Training

Each subject underwent acoustic training in the VR environment in order to get familiar with the equipment and task, and to reach a baseline localization performance (Middlebrooks, 1999). The training consisted of 300 trials with a 2,000-ms white noise burst spatialized from a direction randomly selected from a uniform distribution over the available directions of the interpolated HRTF set.

At the start of each trial, the subjects oriented their head straight ahead by placing a cross-hair over a visual target at (0°, 0°) presented *via* the HMD. The stimulus was then played, during which the subjects kept their head still. At the end of the stimulus, the subjects pointed toward their perceived source direction with a hand-tracking device to provide their localization estimate. Visual feedback was then provided of the actual source direction, and the stimulus repeatedly played until targeted a second time, so that the subjects could familiarize themselves with the available dynamic cues.

#### 2.5.2. Static localization

In the static localization experiment, head movements were restricted by instructing the subjects to keep their head aligned with the reference position. Further, the binaural presentation did not consider subject's head movements. The experiment consisted of nine blocks (three repetitions of three stimulus conditions) of 52 trials, with each trial representing one tested sound direction. The stimulus was a 2,000-ms white noise burst. Within each block, directions were selected from the grid in a random order, while ensuring that each direction was presented once. The order of blocks was ordered randomly across the listeners. No feedback was provided to the subject, neither on the subject's performance nor the actual sound- source positions.

#### 2.5.3. Dynamic localization

In the dynamic localization experiment, subjects were instructed to make a specific one-sided rotation around either the yaw or the pitch axis, as soon as they heard the stimulus onset. At the beginning of each trial, the subject was asked to rotate to the reference position (0 azimuth, 0 elevation) and an arrow was presented on the HMD to instruct the direction of head rotation, indicating the direction of either the pitch axis (up or down), or the yaw axis (left or right). The subject then confirmed to be ready. The head rotation speed was left unrestricted, but was monitored through the tracking system of the VR headset and recorded for the analysis. Then the stimulus was played and the head orientation was recorded. After a head rotation of 10° was registered, the stimulus was again gated off over 5 ms, so that dynamic acoustic cues were only provided for 10° of rotation. Finally, the subject was asked to point to the perceived sound direction and confirm with a press of the button. This process was repeated for 12 blocks (three repetitions for four stimulus conditions). Each block consisted of 208 trials (four arrow directions for 52 source directions). All other details were as in the static-localization task.

Note that this task of dynamic sound localization differed from the head-sweep method used in dynamic sound localization experiments (Macpherson, 2013). In this experiment, the movement was initiated after, not before, the stimulus onset. Therefore, this experiment did not test the isolated dynamic localization performance, but instead tested the added benefit of head movements after a period of static localization.

### 2.6. Data analysis

Before investigating individual localization performance metrics, head orientations recorded during the stimulus presentation (for dynamic and static free-field localization) were checked for outliers. Responses linked with head rotations smaller than 5° were excluded. Responses linked with rotations along the incorrect axis (i.e., axis orthogonal to the instructed axis) of more than 2° were also excluded.

Localization performance was assessed by three metrics; lateral and polar precision errors (LPE and PPE, both in degrees), i.e., standard deviations (Middlebrooks, 1999; Majdak et al., 2010), and front-back confusions (FBC) rate, i.e., each response was labeled by 0 for the correct hemisphere and 1 for a reversal, then the rate (in %) of FBCs was calculated. In identification of front-back errors, target locations with an absolute lateral angle over 60° were ignored, and responses were allowed to cross the midline by 10°. For PPE, the trials that resulted in an FBC were omitted before calculation to keep all metrics independent.

Each subject participated in all experiments, allowing an extensive within-subject analysis. The lateral and polar precision was analyzed using a linear mixed-effects model. FBC data was analyzed with a mixed-effects logistic regression. For both model types, the subject was treated as a random variable. Stimulus type and rotation type, as well as the interaction between the two, were treated as fixed variables. As a follow-up analysis, the estimated (least-squares) marginal means were compared, using Tukey's adjustment for multiplicity. The statistical significance was considered below the levels of *p* of 0.05 as significant and *p* of 0.001 as highly significant. These statistical analyses were run in R version 4.2.1 (R Core Team, 2020).

## 3. Results

### 3.1. Head rotations

Nine thousand nine hundred eighty-four responses were collected each for yaw and pitch rotation. They were checked for the head-movement related outliers. After the clean-up, 8,488 localization responses were obtained for yaw rotation and 8,364 estimates were obtained for pitch rotation. Subjects NH919 and NH1016 showed extremely slow head rotations compared to the other subjects, i.e., almost all trials employed a head rotation speed below 30°/*s*. Still, there was no evidence that justified removing these data.

The maximum velocities of head rotations averaged over all subjects were low (36.41 ± 23.03°/*s* for yaw rotations and 28.54 ± 17.50°/*s* for pitch rotations). Stimulus duration for dynamic listening was on average below one second with the averages of 645.53 ± 447.78 ms for yaw rotations and 802.12 ± 545.91 for pitch rotations.

Finally, reaction times of rotation initiation after the stimulus started were on average in the range between 100 and 200 ms. These reaction times are slightly lower than those found in previous studies, e.g., 200–300 ms in Perrett and Noble (1997a). This can be explained by the button press before each trial, which may have primed the subject to be more responsive to the next stimulus.

### 3.2. Localization performance

**Figure 3:**
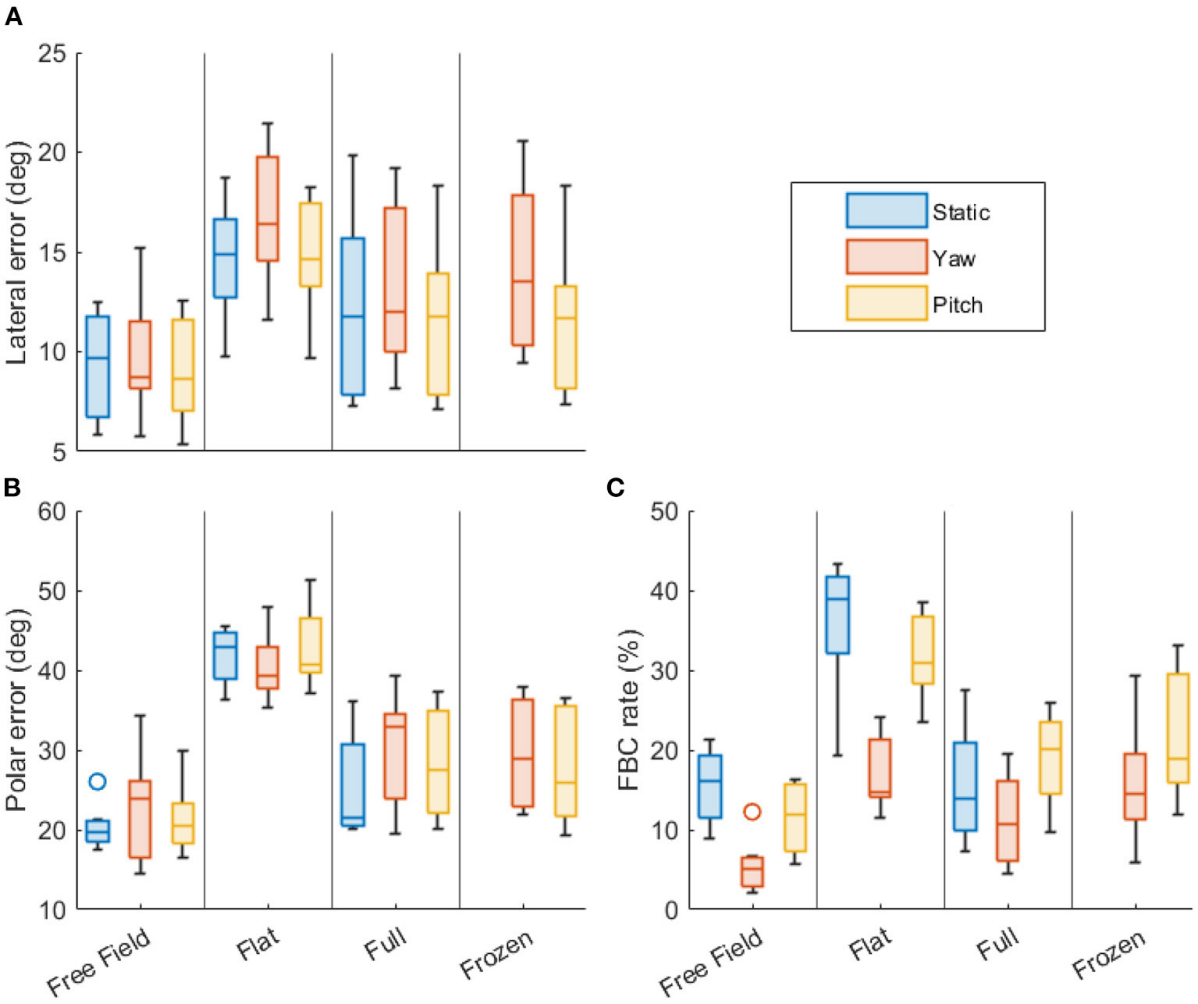
Localization performance grouped by the available acoustic cues. **(A)** Lateral precision error (in degrees). **(B)** Polar precision error (in degrees). **(C)** Front-back confusion rate (in %). Lower values indicate better performance. Each boxplot shows the statistics of all subjects (median, first and third quartiles, minima and maxima, outliers).

**Figure 4:**
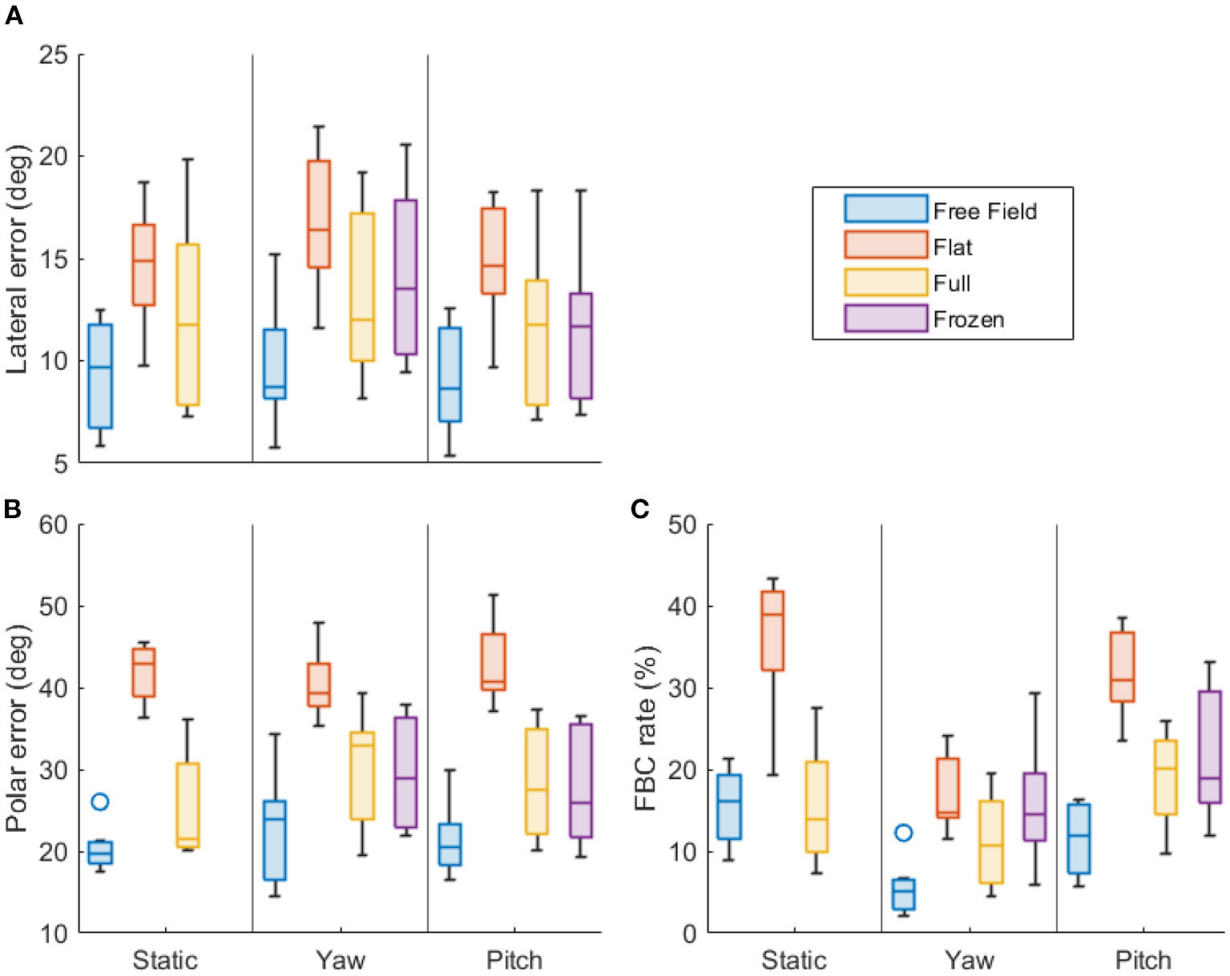
Localization performance grouped by the type of head rotation. **(A)** Lateral precision error (in degrees). **(B)** Polar precision error (in degrees). **(C)** Front-back confusion rate (in %). Lower values indicate better performance. Each boxplot shows the statistics of all subjects (median, first and third quartiles, minima and maxima, outliers).

Figures 3, 4 show the statistics of the localization performance of all subjects. Both figures present the same data, but they are grouped differently for easier comparison across the types of head rotation (Figure 3) and the acoustic cues (Figure 4). Boxes show the lower and upper quartiles with the median represented by the line in the center, whiskers show the non-outlier minima and maxima, and the small circles show the outliers.

Figure 3 facilitates the comparison across the types of the head rotation by comparing across the rotation conditions, encoded by color, within each stimulus condition. Each column shows the performance metrics for no movement (blue), yaw rotation (red), and pitch rotation (yellow), grouped by the available acoustic cues. Results of the statistical analysis of the differences across the types of head rotation (per acoustic cue) are presented in Table 1.

**Table 1:** Statistical significance of differences in three localization performance metrics (lateral error, polar error, FBC rate) between the tested types of head rotation, grouped by the type of acoustic cues.

**Cue**	**Tested rotation**	***p* lat err**	***p* pol err**	***p* FBC**
Flat	Static/yaw	0.6640	0.1172	**< 0.0001**
Flat	Static/pitch	0.9999	1.0000	0.8634
Flat	Yaw/pitch	**0.0007**	0.1979	**< 0.0001**
Full	Static/yaw	0.9600	0.8780	0.2511
Full	Static/pitch	0.9980	0.9988	0.6090
Full	Yaw/pitch	**0.0073**	0.9999	**0.0010**
Free field	Static/yaw	1.0000	0.6076	**< 0.0001**
Free field	Static/pitch	1.0000	0.9604	0.4239
Free field	Yaw/pitch	0.8441	1.0000	**0.0001**

Statistically significant results are printed in bold.

Figure 4 facilitates the comparison across the available acoustic cues: Each column shows the performance metrics for the conditions “flat” (dITD, blue), “full” (dITD and dMSS, red), “free field” (natural listening with all cues, yellow), and “frozen” (dITD with MSS only, violet), grouped by the type of the head rotation. Results of the statistical analysis of the differences resulting from the available acoustics cues (per type of the head rotation) are presented in Table 2.

**Table 2:** Statistical significance of differences in three localization performance metrics (lateral error, polar error, FBC rate) between the tested acoustic cues, grouped by the type of head rotation.

**Rotation**	**Tested cue**	**p lat err**	**p pol err**	**p FBC**
Static	Full/flat	0.2540	<**0.0001**	<**0.0001**
Static	Full/free field	0.3313	0.2498	1.0000
Yaw	Full/flat	**0.0227**	**0.0089**	0.0868
Yaw	Full/free field	0.0810	0.2736	**0.0001**
Yaw	Full/frozen	0.9936	1.0000	**0.0098**
Yaw	Frozen/flat	0.2236	**0.0093**	0.9997
Pitch	Full/flat	0.0684	**0.0002**	**0.0014**
Pitch	Full/free field	0.4723	0.3065	**0.0002**
Pitch	Full/frozen	1.0000	1.0000	0.9129
Pitch	Frozen/flat	0.1327	**0.0001**	**0.0085**

Statistically significant results are printed in bold.

## 4. Discussion

### 4.1. General

As a first check, subject performance in conditions already tested in previous studies was analyzed, with the goal to verify the general quality of this experiment's sound presentation. For the static listening situation in the free field, average localization performance was 9.3°, 20.4°, and 15.4% (LPE, PPE, and FBC rate, respectively). This is similar to the performance usually found in such experiments (e.g., 10.6°, 22.7°, 4.6% in Middlebrooks, 1999), with exception of the FBC rate. The larger FBC rate is surprising, however, it was not problematic for the experiment because it prevented floor effects by providing room for improvements when testing conditions including head rotations. The increased FBC appears to be related to the definition chosen to categorize FBCs. Indeed, when applying the FBC definition provided by Middlebrooks (1999) the average FBC rate for static listening in the free field is 6.3%.

The comparison between the free-field and full conditions sheds light on the quality of the binaural rendering. In the static full condition, the performance was 12.0°, 25.7°, and 15.1% (LPE, PPE, and FBC rate, respectively), which is a small but not significant difference to the static free-field condition (compare row #2 in Table 2). This implies that the HRTF measurements, HRTF manipulations, and the static binaural spatialization adequately simulated the loudspeaker-based reproduction. The localization performance in the full condition was in the range (again excluding FBC rate) of that usually found in such experiments (e.g., 14.5°, 28.7°, and 7.7% in Middlebrooks, 1999; 13.6°, 21.9°, and 12.8% in Majdak et al., 2011, calculated based on Majdak et al., 2010). This indicates that the subjects here localized static sounds with the precision usually found in the literature.

In the dynamic conditions (yaw and pitch), no significant differences were found in the localization precision (LPE and PPE) between the free-field and full conditions, indicating that the general presentation of dynamic cues worked as expected. However, there was a significant difference in the FBC rates (compare rows #4 and #8 in Table 2) and from the free-field condition to the full condition, the FBC rates increased from 5 and 11% (yaw and pitch, respectively) to 10 and 20%. This implies that the dynamic spatialization may have had some short-comings.

No differences were found between full and free-field FBC rates in the static condition, nor were found in the localization precision. This suggests that the increased FBC rates in the dynamic conditions probably weren't caused by problems in static MSS and ITD cues created by imperfections in the HRTF interpolation or differences between headphone and loudspeaker coloration. Artifacts in dITDs might have been the origin, though, for example because of a too large head-tracking latency, causing a lag between the actual subject's head position and not-yet correctly spatialized sound, manifesting itself in a slightly increased uncertainty about the plane of the sound direction, reflected in the increased FBC rates. While this problem may play a role in consumer spatialization products, there is no evidence that it affects the interpretation of the experimental results. Most importantly, none of the subjects reported about dizziness, audio glitches, or loss of externalization, which would indicate more serious problems with the dynamic sound reproduction.

A final important observation is the large variance across subjects in some of the conditions, indicating large inter-subject differences in the localization performance. An analysis of the listener variability may provide valuable insights. It is, however, beyond the scope of this study.

### 4.2. Effect of the head rotations

In the free-field condition, there was no significant effect of head rotation on localization precision, neither on LPE nor on PPE. This condition best represents natural listening, suggesting that small head rotations in general do not affect the localization precision. The lack of an effect on precision is consistent with McAnally and Martin (2014), who also showed that rotation does not affect lateral error, and that elevation error only decreases for rotations of 16° and larger. Previous studies that investigated larger pitch rotations than the ones produced here also found no reduction in elevation errors (Thurlow and Runge, 1967; Kato et al., 2003). In the flat and full conditions, a significant effect of head rotation was found, but only when comparing the LPE between yaw and pitch rotations (see rows #3 and #6 in Table 1). These LPE differences were ~2° (see Figure 3A), thus rather small. Interestingly, the LPEs were larger in the yaw conditions indicating that the subjects had more difficulties to localize sound in the lateral dimension when rotating their head along that dimension. This was only the case in the binaural reproduction. This small but significant effect might be related to some issues with the dynamic cue presentation in the study's binaural reproduction. As the lateral localization performance is usually associated with ITD cues, this issue might indicate an imperfection in the ITD and dITD cues reproduction. Note that PPE did not show a significant difference between rotation conditions in any of the cue conditions. This indicates that imperfections with respect to spectral cues such as MSS and dMSS in the binaural reproduction were absent or imperceivable to the subjects. The absence of an effect on PPE with head movements also suggest that small head movements do not induce the so-called Wallach cue, i.e., the rate of change in source azimuth angle relative to the change in head orientation, which has been hypothesized to provide information on elevation angle (Wallach, 1940; Perrett and Noble, 1997b). The general lack of an effect of head rotations on localization precision shows that the information gained from dynamic cues due to small head rotations is either too small to improve the precision or, alternatively, is canceled out by the increased uncertainty caused by head rotation. Both hypotheses align well with findings that lateral and polar errors decrease for head rotations larger than 45° only (McAnally and Martin, 2014).

For the FBC rates, yaw rotations yielded reduced rates in all cue conditions, with the flat and free-field conditions showing a highly significant decrease of ~25 and 10%, respectively (see Figure 3C). This shows that small yaw rotations effectively aided in resolving FBCs, even when all acoustic cues were available. This is consistent with Wallach (1940) and Thurlow and Runge (1967). Pitch rotations, on the other hand, did not yield any significant changes in the FBC rates in any stimulus conditions, indicating that pitch rotations do not help in resolving front from back.

Taken together, these results demonstrate that small head rotations do not improve localization precision, however, yaw rotations, even as small as 10°, do help listeners to resolve front from back. This benefit is, most clearly visible in conditions without MSS cues (flat), but remains present even in the free field, when the MSS cues are available. This means that dITD is still informational in the presence of MSS cues, and thus is not redundant. As hypothesized, pitch rotations do not affect the sound localization performance at all. Pitch rotations follow the interaural axis, thus do not induce any dITD or dILD cue, leaving the dMSS as the only available dynamic cue. The lack of an effect caused by pitch rotations indicates that the subjects were unable to utilize dMSS in the present sound-localization task. The effects of the dMSS and other cues are investigated in the following section.

### 4.3. Effects of the acoustic cues

For the lateral precision, LPEs in the flat condition were significantly larger than the full condition during yaw rotation (see row #3 in Table 2). This suggests that, for some subjects, the MSS or dMSS cues in the full condition may have provided additional information that improved lateral precision. There is, however, little evidence from literature that MSS cues contribute to the localization of static sounds along the lateral angle. On the contrary, MSS cues have been rather found as uncorrelated with the localization along the lateral angle (Macpherson and Middlebrooks, 2002). Furthermore, it is also unlikely that dMSS caused this improvement, as no difference was found between the static and the yaw movement in the full-spectrum condition. However, the significant error increase might have originated from two effects adding up: unnatural static ITD-ILD combinations (which alone increased the errors a little but did not cause any significance), and a potential conflict in the dynamic ITD-ILD pairs. The full condition provided natural ITD-ILD pairs, but the flat condition, because of the spectral flattening, might have reduced the ILDs, resulting in unnatural ITD-ILD pairs, yielding to a worse precision in localizing sounds along the lateral dimension. In the static and pitch conditions there was no significant increase in LPEs, indicating that if a potential conflict in the unnatural static ITD-ILD combinations affected the localization then it was not much.

For the polar precision, significant differences were found between the flat and full conditions across all types of rotations (see rows #1, #3, and #7 in Table 2). For the static condition, this is not surprising, as MSS cues are essential for localization along the sagittal planes (Kistler and Wightman, 1992; Baumgartner et al., 2014). Similarly for pitch rotations, which did not provide dITD or dILD cues, this indicates that MSS cues help in localizing sounds along the polar dimension. Note that this benefit is not necessarily evidence for the use of dMSS cues, as the improvement is similar to that in the condition without head movement. For the yaw rotation, the differences between flat and full conditions indicate that MSS cues provided additional information on the polar angle, despite the availability of dITD and dILD cues.

The analysis of the frozen condition can further help in disentangling the contribution of the MSS and dMSS cues because the frozen condition provided the actual ITD and dITD cues, but the MSS cue was “frozen” to that of the initial head orientation, thus provided no dMSS cue. In the frozen condition, the PPEs were at similar levels for both types of rotation (see Figure 4B) and there was no significant difference between the full and frozen conditions (see rows #5 and #9 in Table 2). This indicates that the absence of the dMSS cue in the frozen condition was not relevant for the localization precision in the polar dimension. When compared to the flat condition, however, the frozen condition yielded significantly improved localization precision (see rows #6 and #10 in Table 2), indicating that the static MSS cues were essential for the polar localization precision even in the presence of head rotations.

For the FBC rates, in the conditions without head rotations, there was a significant difference between the full and flat conditions, confirming the clear contribution of MSS cues when resolving front from back in a static sound-localization task (Langendijk and Bronkhorst, 2002). In the conditions with pitch rotations, there was a significant difference between the conditions full and flat, but there was no significant difference between the conditions full and frozen (see rows #7 and #9 in Table 2). This demonstrates that MSS cues, but not the dMSS, were essential to resolve front from back even when pitch rotations were involved. For the yaw rotations, the situation was different. No statistically significant differences were found between the flat and full conditions. This suggests that neither MSS nor dMSS cues provided a benefit to resolve front from back when the subject were allowed to rotate the head along the yaw axis. Thus, small head rotations seem to sufficiently have compensated for the absence of any spectral cues.

Next, a small but significant difference was found between the full and frozen conditions for the yaw rotation. The hypothesis behind this finding might be the effect of the ILD cue, which remained constant over the course of the frozen stimulus, thus potentially creating a conflict between the dynamic interaural cues, i.e., dILD and dITD. These potential conflicts in the frozen condition may have slightly but significantly reduced the ability to resolve front from back. Note that such difference has not been found for pitch rotation, probably because the change of the interaural cues over this axis is minimal. This hypothesis is supported by findings of dILD as a perceivable cue, though it is mostly weaker than dITD cues (Pöntynen and Salminen, 2019). Experiments on conflicting dynamic cues, such as the “Wallach illusion” (Macpherson, 2011; Brimijoin and Akeroyd, 2012; Pöntynen et al., 2016) or the “phantom walker illusion” (Martens et al., 2013), have previously shown that conflicting dynamic interaural cues can be a cause for unstable location perception. Such illusions seem to be strongest when high-frequency spectral cues are absent, i.e., the spectral cues can correct for a conflicting dITD. Note that these studies did not address dITD vs. dILD cues or the dynamic aspect of spectral cues explicitly. On the other hand, in another study involving an n0sπ task it was found that in the presence of conflicting dILD and dITD cues the dITD cues dominated for binaural signal detection (van der Heijden and Joris, 2010). However, it is not clear whether this result can be extrapolated to a dynamic localisation task as considered here.

The benefit of dynamic monaural spectral cues for sound localization has been rarely discussed. A reduced elevation error has been observed for large pitch rotations during stimulus presentation (McAnally and Martin, 2014), suggesting an effectiveness of dynamic spectral cues with head rotation. Often, however, pitch rotation did not produce an improvement in localization performance (Thurlow and Runge, 1967; Kato et al., 2003). In a monaural listening experiment, normal-hearing listeners with one ear plugged showed no benefit of head rotations when localizing sounds located in the horizontal plane (Hirahara et al., 2021). However, single-sided deaf listeners do appear to utilize and even rely on changes in head position to induce changes in the monaural cues produced by the direction-dependent high-frequency attenuation resulting from acoustic head shadowing (Pastore et al., 2020).

Taken together, these results demonstrate the important contribution of static MSS cues to sound localization performance, even during sound localization involving head movements. The results also suggest that, for small head movements, dynamic changes of that cue are not evaluated by the auditory system. This has a direct implication for modeling active sound localization in human listeners. Recently, a model of active sound localization based on Bayesian inference was proposed (McLachlan et al., 2021). That model only implemented the dITD as an additional dynamic cue, though the necessity for the consideration of other cues was at that point unclear. As it seems, further updates of the MSS cues (dMSS cues) are not required when modeling human sound localization, at least for small head movements.

## 5. Conclusions and future work

In this study, the influence of small head movements on localization performance was investigated by means of three metrics. The results show no additional benefit of small head rotations (up to ±10°) on lateral and polar localization precision. Only yaw rotations significantly reduced the front-back confusions, whereas pitch rotations were of no help. This finding could be explained by the contribution of dynamic ITD cues (and, to a lesser extent, the dynamic ILD cues). These effects were most prominent for stimuli devoid of monaural spectral cues, but remained even when these cues were available. The analysis of the frozen spectrum condition, which provided the actual static and dynamic ITD cues, but “froze” the monaural spectral cues to those from the initial head orientation, suggests that humans do utilize the static monaural spectral cues but are insensitive to their dynamic changes over the course of the stimulus. This is clearly supported by the results showing that monaural spectral cues, fixed to those from the initial head orientation and conflicting with the dynamic ITD cues, did not impair the localization performance.

There are several directions that future work can move toward. First, due to the differences between the binaural and loudspeaker performance in the dynamic listening conditions, it would be insightful to compare various loudspeaker-based conditions by using, for example, band-pass filtered instead of broadband signals. Second, a direction-dependent analysis of the localization performance may reveal which spatial regions gain from the head movements most. Third, the results showed a large inter-subject variability. Thus, a listener-specific analysis of the contribution of static and dynamic cues may be interesting. A similar analysis has been done for the acoustic and non-acoustic factors contributing to the localization performance in sagittal planes (Majdak et al., 2014). Finally, these findings will help to further develop the previously proposed model of active directional sound localization (McLachlan et al., 2021).

## Data availability statement

The raw data supporting the conclusions of this article will be made available by the authors, without undue reservation.

## Ethics statement

Ethical review and approval was not required for the study on human participants in accordance with the local legislation and institutional requirements. The patients/participants provided their written informed consent to participate in this study.

## Author contributions

GM, PM, JR, and HP contributed to conception and design of the study. MM and PM set up the experimental software. MM conducted the experiments. GM performed the statistical analysis and wrote the first draft of the manuscript. HP, PM, and MM wrote sections of the manuscript. All authors contributed to manuscript revision, read, and approved the submitted version.
